# Multiphoton Microscopes Go Big: Large-Scale In Vivo Imaging of Neural Dynamics

**DOI:** 10.34133/2022/9803780

**Published:** 2022-07-26

**Authors:** Janelle M. P. Pakan, Yuguo Tang

**Affiliations:** ^1^Institute for Cognitive Neurology and Dementia Research, Otto von Guericke University, Magdeburg 39120, Germany; ^2^Suzhou Institute of Biomedical Engineering and Technology, Chinese Academy of Sciences, Suzhou 215163, China

Ever since Santiago Ramon y Cajal’s pioneering observations of precise neuronal structure, neuroscience has been devoted to deciphering how these individual brain cells engender the complexity that defines brain function. Understanding the fundamental processes of perception, cognition, and behavior through neural activity remains a major challenge in modern neuroscience. Considering the size and number of neurons in the brain, as well as the distributed nature of neural activity across interconnected networks, it is increasingly obvious that we need advanced systems to directly measure neural activity in real time to assess both coordinated activity on a large scale and the brain’s high degree of specialization on a small scale. In addition to histological and in vitro studies, examining the living brain is also vital for a full understanding of brain function. The combined need for multiscale approaches and in situ evaluation of neuronal activity has led to rapid technological advances for the field of *in vivo* microscopy (for review, see [1]).

Over the last decade, the number of neurons that can be imaged near simultaneously while maintaining single-cell resolution has increased exponentially in rodent models—climbing from tens of neurons in the earliest studies to current limits exceeding one million neurons [2]. Several novel approaches using two-photon microscopy have contributed to this recent escalation. For instance, while two-photon microcopy traditionally relies on point-by-point scanning across a given limited area, mesoscale microscopy (i.e., imaging with subcellular resolution and fields of view spanning several millimeters) has been achieved using advances in scanning techniques. Fast three-dimensional scanning [3] and dual scanning [4] systems with appropriately scaled optics have allowed scientists to image tens of thousands of neurons within a larger imaging volume across multiple brain regions. However, while these approaches enable multiarea imaging by rapid switching between brain regions, they are limited in either moving between brain regions laterally (across the surface of the brain) or axially (along the depth of the cortex), but not easily along both dimensions. Because of the curvature of the cortex, sampling anywhere along both the lateral and axial planes would allow for more flexibility in targeting both multiple cortical regions and across various cortical layers of interest.

Beyond the cortex, there is also a growing demand to monitor brain-wide neural activity simultaneously. While methods such as functional magnetic resonance imaging (fMRI) and the more recently developed ultrasound imaging techniques can provide whole-brain proxy readouts of neural activity at increasingly impressive resolution in rodents (e.g., <200 *μ*m resolution achieved with ultrahigh field 9.4 tesla fMRI), these methods, relying on changes in cerebral blood flow, will never approach the spatial or temporal precision needed to read out single-cell activity. Similarly, electrophysiological techniques still provide the gold standard for recording neuronal activity with high temporal precision; however, the capacity to probe complex cell-type-specific circuit dynamics remains limited. Therefore, optical approaches using multiphoton microscopy remain highly advantageous because of the optimal balance between the temporal and spatial resolution that can be achieved using these methods. When the capacity for multiarea optical imaging is expanded beyond a single plane of the accessible cortical surface, the strength of these methods is further increased. One strategy for achieving this is to use a compound objective assembly to distribute one light path across multiple imaging planes, which can be flexibly placed across both the lateral and axial dimensions [5]. Using this strategy has allowed for simultaneous imaging of single-cell resolution neuronal activity across the visual cortex, the motor cortex, and the hippocampus [5]. However, while flexible, the individual fields of view are still limited to a single imaging plane using this approach—ultimately constraining the total number of neurons that can be imaged from each brain region.

The current upper limit of near simultaneously imaged neurons has sampled over one million cells using light bead microscopy [2]. This approach harnesses the pulse rate of a two-photon laser to generate interleaved pulses that are focused across different axial positions (i.e., across the tissue depth). In this way, 30 imaging planes can rapidly move through the tissue to perform mesoscale volumetric imaging with single-cell resolution [2]. So far, this technique has been limited to probing cortex-wide neuronal activity, but the combination of this approach with compound objective imaging techniques [5] offers exciting possibilities for large-scale, brain-wide, single-neuron resolved imaging.

It is particularly important that these approaches can be used to measure neural activity in behaving animals. In this regard, *in vivo* microscopy also offers advantages over fMRI and other more restrictive neuroimaging techniques. Additionally, two-photon microscopy remains the only reliable method for chronically observing the same neurons over days, weeks, and even months. This is vital for studies examining the underlying mechanisms of plasticity during learning and to investigate fundamental principles of memory, including the formation of memory engrams. As *in vivo* microscopy continues to push the technological boundaries, advances in biological sensors (e.g., voltage-based and neurotransmitter sensors) and ever-improving optogenetic tools for circuit manipulations will also expand the repertoire of experimental questions that can be addressed using optical technologies. With these advancements, we will be better equipped to probe and analyze the brain’s inner workings.
